# A DLC-Punch Array to Fabricate the Micro-Textured Aluminum Sheet for Boiling Heat Transfer Control

**DOI:** 10.3390/mi9040147

**Published:** 2018-03-25

**Authors:** Tatsuhio Aizawa, Kenji Wasa, Hiroshi Tamagaki

**Affiliations:** 1 Department of Materials Science and Engineering, Shibaura Institute of Technology, 3-9-14 Shibaura, Minato-City, Tokyo 108-8548, Japan; 2 TECDIA, Co., Ltd., 4-3-4 Shibaura, Minato-City, Tokyo 108-0023, Japan; k_wasa@tecdia.co.jp; 3 NIRO, Co., Ltd., 6-1 Minatojima-Nakamachi, Chuo-Ward, Kobe 650-0043, Japan; tamagaki@niro.or.jp

**Keywords:** plasma printing, multi DLC-punch assembly, plasma oxidation, micro-cavity textures, micro-embossing, aluminum sheets, boiling heat transfer

## Abstract

A diamond-like carbon (DLC) film, coated on an SKD11 (alloy tool steel) substrate, was shaped by plasma oxidation to form an assembly of DLC macro-pillars and to be used as a DLC-punch array that is micro-embossed into aluminum sheets. First, the SKD11 steel die substrate was prepared and DLC-coated to have a film thickness of 10 μm. This DLC coating worked as a punch material. The two-dimensional micro-patterns were printed onto this DLC film by maskless lithography. The unprinted DLC films were selectively removed by plasma oxidation to leave the three-dimensional DLC-punch array on the SKD11 substrate. Each DLC punch had a head of 3.5 μm × 3.5 μm and a height of 8 μm. This DLC-punch array was fixed into the cassette die set for a micro-embossing process using a table-top servo-stamper. Furthermore, through numerically controlled micro-embossing, an alignment of rectangular punches was transcribed into a micro-cavity array in the aluminum sheet. The single micro-cavity had a bottom surface of 3.2 μm × 3.2 μm and an average depth of 7.5 μm. A heat-transfer experiment in boiling water was also performed to investigate the effect of micro-cavity texture on bubbling behavior and the boiling curve.

## 1. Introduction

Carbon-based materials have been utilized in various applications. Diamond-like carbon (DLC) and diamond films are coated onto metallic substrates and silicon wafers as protective coatings or as structural layers [1]. Carbon nanotubes as well as graphene films are also used [2]. Such carbon-based materials have industrial applications for their marginable electrical resistivity and thermal conductivity, high strength and hardness, chemical inertness, environmental friendliness, etc. [3].

Shaping these carbon-based films into functional components with complex geometry has become important in engineering [4]. Since a DLC film has high hardness and low ductility, mechanical machining, electrically discharging, and laser machining cannot be performed to shape these carbon-based materials. The authors have been concerned with plasm oxidation printing to fabricate multi-DLC-punch assembly dies for micro-stamping [5,6,7]. In their studies, DLC-punch height was limited to a film thickness of 5 μm. The authors, using plasma enhanced chemical vapor deposition (PECVD), succeeded in extending the DLC film thickness up to 20–30 μm [8]. Plasma oxidation printing is now used to transform an ink-jet printed micro-patterns into micro-textured DLC films with heights from 10 μm to 20 μm [9,10]. The spatial resolution here is governed by the viscosity and stability of the heat-resistant ink. An alternative micro-patterning process is needed to improve the spatial resolution. Many studies have reported on, in addition to the micro-texturing of DLC films, the formation of micro-/nano-pillars into semi-conductive materials [11]; however, such methods are not suitable for building micro-pillars on large substrate surfaces. In particular, a fine micro-pillared punch is necessary to fabricate micro-cavity metallic sheets for engineering applications.

In the present study, maskless lithography was employed to print an initial two-dimensional micro-pattern with a spatial resolution down to 1 μm onto a DLC-coated die substrate. First, a thick DLC coating was deposited via PECVD onto steel substrates with thicknesses of 10 μm. This micro-patterned substrate was plasma-oxidized to selectively remove unprinted DLC films and to form a micro-textured DLC punch. This DLC punch was fixed onto a cassette die for a micro-embossing process with use of the computer numerical control (CNC) stamping system. Through this process, the micro-pillar textures on the DLC punch were transcribed into micro-cavity textures on an aluminum sheet. A single rectangular DLC-pillar punch can form a rectangular micro-cavity into the aluminum sheet. 

Boiling heat-transfer experiments were performed to investigate the effect of micro-cavity-textured aluminum on the boiling curve as well as the micro-bubbling behavior in the boiling on aluminum sheets. As pointed out in [12], a micro-wedge naturally formed on the heated metallic sheet works as a bubble nucleus. As the superheat in a boiling curve is increased, many vapor bubbles on a metallic sheet nucleate, grow by themselves, and coalescence to form a vapor film at the burn-out point. This conventional boiling heat transfer is necessarily affected by the presence of micro-cavity textures with a size equivalent to that of a naturally formed bubble nucleus. Most previous studies [13] on the effect of micro-textures on boiling heat transfer have utilized silicon technology to fabricate micro-cavity textures on silicon wafers. A micro-cavity-textured metallic sheet is necessary to observe the direct impact of preexisting micro-cavity textures on the boiling heat transfer of thermally conductive metals. In the present experiment, the micro-embossed pure aluminum sheet with a size of 10 mm × 30 mm was employed as a metallic solid surface in the heat transfer to water. The observed bubbling behavior on the bare and textured aluminum sheets demonstrates the micro-cavity effect on boiling. The measurement of the boiling curve, by varying the superheat, shows the difference in heat transfer between two aluminum sheets. 

## 2. Experimental Procedure

In this section, the plasma oxidation printing procedure is introduced with comments on the computer-aided design (CAD) data preparation for maskless micro-patterning. The high-density plasma oxidation system, a key technology for precisely shaping DLC films coated on steel substrates, the micro-embossing system, utilized as a tool to transcribe the micro-pillar texture to the micro-cavity texture in the metallic sheet, and the heat transfer experiment procedure, used to investigate the effect of micro-cavity textures on the boiling heat transfer in the channel flow, are then described.

### 2.1. Plasma Oxidation Printing

Plasma oxidation printing consists of two steps: maskless micro-patterning, with use of lithography, and high-density plasma oxidation. The original two-dimensional micro-pattern was data-mined for printing; if needed, these subsets of CAD data were duplicated to prepare the data for printing the entire substrate surface. The final CAD data were transferred into the maskless lithography to print the initial micro-pattern onto the DLC film directly. Both the nano-carbon resist and the platinum deposits were employed to print the thermal-resistant micro-pattern. This micro-patterned DLC film was plasma-oxidized to selectively remove the unprinted DLC parts from the original DLC film. Finally, this DLC punch was processed to remove residual deposits and resists before the DLC-punch surface was finished and polished for a maximum surface roughness of 0.1 μm.

### 2.2. Maskless Micro-Patterning

**Figure 1:**
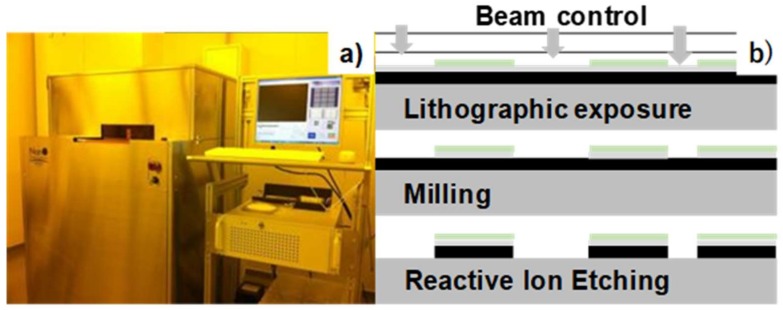
The maskless micro-patterning process. (**a**) Outlook of the maskless lithography system. (**b**) Three steps for two-dimensional micro-patterning.

The maskless micro-patterning method via lithography was utilized to print the initial two-dimensional micro-patterns directly onto the DLC surface. Figure 1a depicts the maskless lithography equipment with which lithographic exposure takes place with positioning control using the prescribed CAD data. The present maskless micro-patterning procedure consists of three steps as shown in Figure 1b: maskless exposure by the beam control, a milling process to remove the platinum deposit, and reactive ion etching to remove nano-carbon resin. The spatial resolution in this micro-patterning process is limited to 1 μm. The larger surface of the DLC punch is micro-patterned by copying the CAD data. The original CAD data for micro-patterning onto surfaces of 10 mm × 10 mm is copied twice to prepare the CAD data and to enable micro-patterning onto surfaces of 10 mm × 30 mm. 

### 2.3. The Plasma Oxidation System

**Figure 2:**
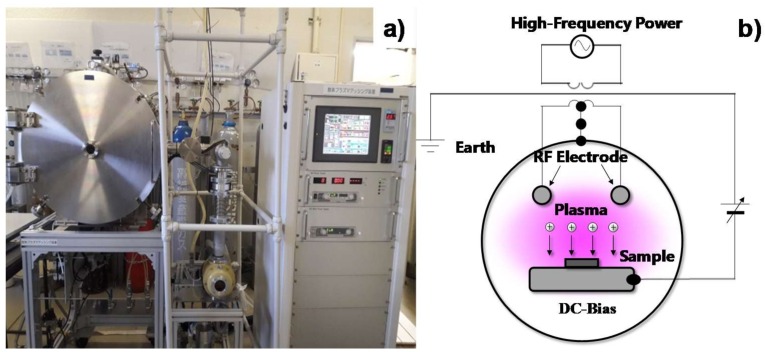
The plasma oxidation process. (**a**) Outlook of the plasma oxidation system. (**b**) Illustration of the experimental setup.

This system works using radio frequency (RF) and direct current (DC) plasmas to etch and ash carbon-based materials, including DLC films. In particular, a hollow cathode device is utilized to intensify the ion and electron densities so as to activate a plasma-chemical reaction between the carbon in the DLC component and the oxygen species in the plasmas. As shown in Figure 2, this system consists of five main parts; e.g., the vacuum chamber, the evacuation pumping unit, the RF/DC power generators, the RF controller, and the control panel. Both the RF power and the DC bias were controlled independently to search for the suitable plasma conditions for the oxidation process. In the experiments, the chamber was evacuated to the base pressure after specimens were placed in it. Then, the oxygen gas was introduced into the chamber at a constant pressure of 40 Pa. The plasma oxidation was performed for 3.6 ks under an RF voltage of 250 V and a DC bias of −500 V.

### 2.4. The Micro-Embossing System

As explained in detail by [14,15], the CNC-stamping system with a maximum load of 200 kN was employed for micro-embossing the pure aluminum sheet with a thickness of 0.2 mm. In the present CNC-stamping system with a feeder and a cropper, both loading and unloading steps as well as feeding and cropping movements worked also via CNC control. The loading and unloading sequences were directly controlled with a PC. In the stamping test, the lower die was fixed onto the lower bolster of stamper. Only the upper die was moved down and up for loading and unloading, respectively, in a time sequence. Both the applied load and the stroke of the upper die were monitored online via the load cell and the linear scale, respectively. After analyses in [16,17], the pulsewise motion was employed to control the loading and unloading processes in the following micro-embossing process.

### 2.5. Heat Transfer Experiments

Based on the literature on boiling heat transfer [12,13,18], water transforms to vapor, and the vapor bubbles nucleate and grow themselves in the cavities on the heated metal surface. Hence, the heat transfer process from metallic solid to water inevitably accompanies the nucleation and growth of vapor bubbles swelling on the metallic surface. 

**Figure 3:**
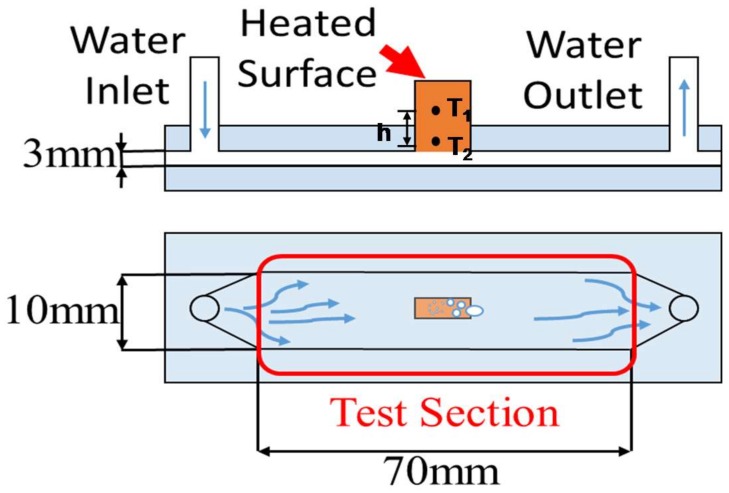
Experimental setup for heat transfer measurement and for observation of the bubbling during the heat transfer in boiling.

The heat-transfer in boiling pure water was measured to investigate the boiling curve between the heat flux and the superheat, and to observe the bubbling behavior on bare and micro-textured aluminum sheets. Figure 3 illustrates the present experimental set-up. The pure water was flown from the inlet to the outlet of the channel through the test section with a constant subcooled temperature of 30 K. The bare and micro-cavity-textured aluminum sheets with sizes of 10 mm × 30 mm × 0.2 mm were conjoined to the copper support block. The aluminum surface temperature (T_s_) was controlled to heat up the copper block. The superheat, ΔT_sat_, was obtained by the difference between the measured T_s_ and the saturated temperature of the pure water. Through the thermo-couples embedded into the copper block, the heat flux, q, was also in situ measured in parallel with the measurement of T_s_. The difference in the measured heat flux through the aluminum sheet as well as the bubbling behavior to be observed came from the difference in the surface profile of the aluminum sheets; i.e., the bare flat surface or the micro-cavity surface. Since the time resolution is 1 ms, the swelling and moving bubbles could be directly monitored throughout the experiments. 

## 3. Experimental Results and Discussion

The thick DLC-coated SKD11 substrate with a size of 100 mm × 100 mm was fabricated via PECVD and shaped into a DLC-coated SKD11 die with a size of 10 mm × 80 mm by precise machining. The rectangular micro-dot pattern was printed using maskless lithography. This DLC die was further plasma-oxidized to fabricate the starting micro-pillared DLC-punch assembly with a size of 10 mm × 30 mm. This DLC-punch array was fixed onto the cassette die for micro-embossing. The original DLC micro-pillar texture was transcribed onto the pure aluminum sheet to fabricate the micro-cavity-textured sheet. This textured aluminum sheet was utilized in heat transfer experiments to demonstrate that the boiling heat transfer is uniquely affected by the micro-textured aluminum.

### 3.1. The Preparation of Thick DLC Film Deposited SKD11 Substrate

**Figure 4:**
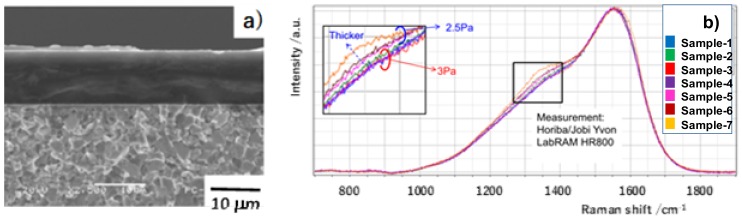
Preparation of the thick diamond like carbon (DLC) coating on the SKD11 substrate. (**a**) Scanning electron microscopy (SEM) image on the cross-section of the DLC-coated SKD11 substrate. (**b**) Raman spectrum of the thick DLC films for various pressures.

The PECVD system with a power of 3 kW and in a middle frequency (MF) of 60–70 kHz was utilized to build the DLC coating with a thickness up to 10 μm on the polished SKD11 substrate. After argon-sputtering and the formation of a metallic interlayer onto the substrate, the main DLC layer was formed using C_2_H_2_ gas as carbon and hydrogen sources of DLC under a pressure (p) of 2.5 Pa. Figure 4 shows a typical cross-sectional scanning electron microscopy (SEM) image and Raman spectra of the seven DLC films deposited by PECVD for 9.72 ks under various pressure levels. The amorphous carbon layer with a uniform thickness was coated on the substrate. G-peak intensity was insensitive to the pressure; D-peak intensity slightly increased with thickness when p was equal to 2.5 Pa, as shown in Figure 4b.

### 3.2. Maskless Patterning onto the DLC Film

**Figure 5:**
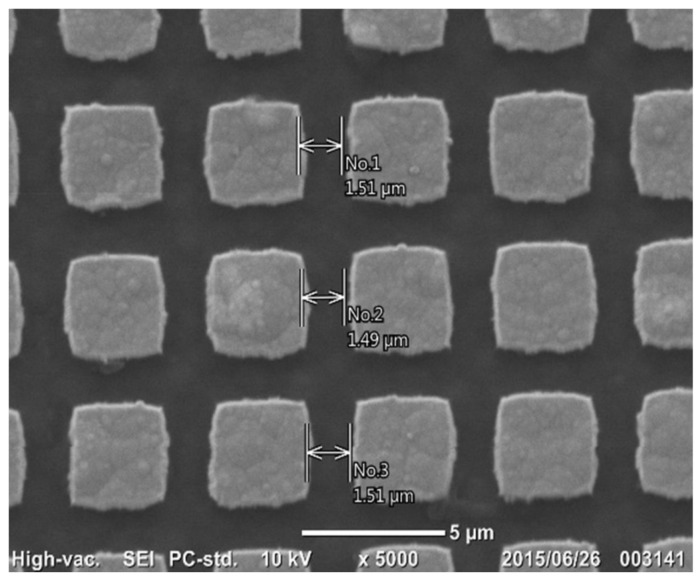
Rectangular micro-dots, printed onto the DLC film surface by maskless lithography.

Maskless micro-patterning by lithography was utilized to print the initial two-dimensional punch-head patterns onto the DLC film surface. Figure 5 depicts rectangular micro-dots, printed on the DLC film with the use of a platinum deposit as well as a nano-carbon resist. A single square micro-dot is 3.5 μm × 3.5 μm in size. The pitch between the adjacent micro-dots is 5 μm. The clearance between two adjacent micro-dots is 1.5 μm. Small deviations that can be seen on the boundaries of the micro-dots come from digital errors in the spatial resolution in the maskless lithography. 

No masks were used in this approach. As shown in Figure 2b, the designed micro-pattern was printed onto the surface of the aluminum deposit by lithographic exposure. Unprinted parts of the aluminum deposits and nano-carbon resists were removed by milling and reactive ion etching, in that order. Various micro-units with geometric complexity were designed via CAD in a straightforward manner and printed onto the DLC films coated on the material substrates. Such maskless micro-patterning is useful for printing any designed micro-head pattern with complex geometries and within a high spatial resolution. In addition, an assembly of micro-punches was also designed and drawn on the DLC film. 

### 3.3. Plasma Oxidation of Micro-Patterned DLC Films

**Figure 6:**
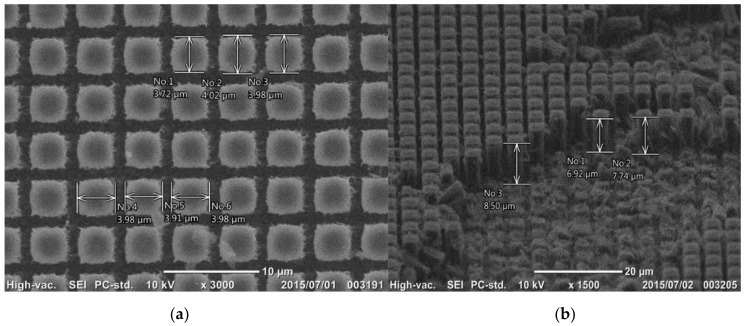
Formation of the DLC micro-punch array by the plasma oxidation process. (**a**) Top view of the micro-pillared DLC punch. (**b**) Cross-sectional view of the micro-pillared DLC punch.

Figure 6 depicts the DLC film subjected to plasma oxidation etching for 3.6 ks. The square micro-dots, printed on the DLC film in Figure 5, worked as a mask to prevent the masked DLC areas from plasma oxidation. The unprinted bare DLC films were selectively etched away; the clearance of 1.5 μm between the square micro-dots in Figure 5 was machined by this plasma oxidation. The rectangular DLC-pillars were formed on the SKD11 substrate from the square micro-dot pattern. The micro-pillar head size was measured based on the SEM top view in Figure 6a. The average head size was estimated to be 3.8 μm × 4.0 μm. Since the initial square micro-dot had a size of 3.5 μm × 3.5 μm and a clearance of 1.5 μm, the machined groove width between the DLC-pillars experienced shortage by 0.3–0.5 μm. As seen in Figure 6a, this shortage came from the irregular head geometry. Figure 6b shows the cross-section of these rectangular DLC-pillars. Although the pillars were damaged during the preparation of the samples for SEM observation, the pillar height reached 8 μm on average after etching for 3.6 ks. The alignment of micro-pillars was observed to have nearly the same clearance of 1.2–1.5 μm; homogeneous etching took place in the present plasma oxidation process. The etching rate was 8 μm/h, which is similar to etching rates recorded in other studies: 10 μm/h in [15] and 9 μm/h in [16].

### 3.4. Micro-Embossing into Aluminum Sheet

**Figure 7:**
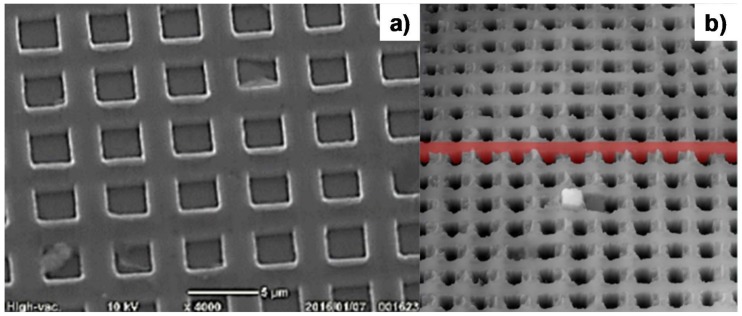
Transcription of the DLC-punch array into pure aluminum sheets by computer numerical control (CNC) micro-embossing. (**a**) SEM image on the micro-cavity textures on the aluminum sheet. (**b**) Laser-microscopic image of the micro-cavity textures.

Through the micro-embossing process via the CNC stamper, the micro-pillar texture on the DLC punch was transcribed into the pure aluminum sheet. The aluminum sheet with a thickness of 0.2 mm was fed from one end and cropped from the other through the die-set. During the micro-embossing process, the tension was applied to this sheet to release the embossed sheet from the dies. As precisely stated in [14,15,16,17], the motion control has a significant influence on the transcription of the micro-texture onto aluminum sheets via the micro-embossing process. Pulse-wise motion with a maximum load of 150 kN and an incremental stroke of 15 kN was employed in the stroke control. Figure 7 shows the SEM image of the stamped aluminum sheet by the micro-embossing process. As depicted in Figure 7a, the DLC-pillar head geometry was transcribed into the bottom of the micro-cavity with an average size of 3.2 μm × 3.2 μm, while the clearance between DLC-pillars was shaped into a micro-cavity wall with a thickness of 1.8 μm. Figure 7b depicts the laser microscopic image of micro-cavity textures into the aluminum sheet. The micro-cavities with an average depth of 7.5 μm were formed with accurate alignment with the pitch of 5 μm. This high-quality transcription into the pure aluminum sheet by the micro-embossing process demonstrates that plasma oxidation can be used to micro-fabricate a micro-pillared DLC-punch array with sufficient accuracy.

### 3.5. Boiling Heat Transfer Testing

**Figure 8:**
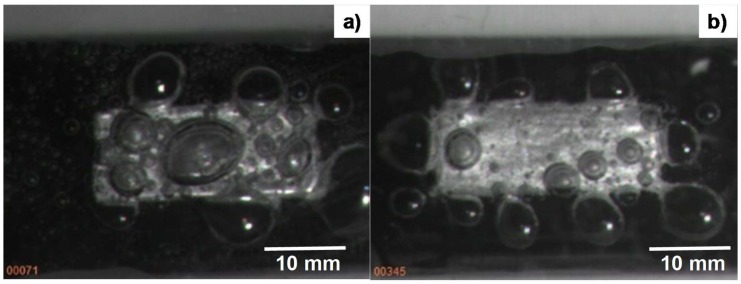
Bubbling behavior on the bare aluminum and on the micro-cavity-textured aluminum when the superheat is 50 K. (**a**) Bubbling on the bare aluminum. (**b**) Bubbling on the micro-cavity-textured aluminum.

Bubbling behavior was first observed and monitored with a video camera with a time resolution of 1 ms. Since both the bare and micro-textured aluminum sheets were fixed on the same copper block, as shown in Figure 3, the difference in the bubbling process between the two comes from the aluminum sheet with and without the pre-existing micro-cavity textures. Figure 8a depicts the bubbles swelling on the flat aluminum sheet fixed on the heated copper block at ΔT_sat_ = 50 K. As predicted in [18], the nucleating bubbles agglomerated into large bubbles and swelled on the flat aluminum sheet. The average bubble size reached 5 mm in diameter. These swelling bubbles on the metallic surface increased in size and finally formed a vapor film that covered the entire metallic surface. At this critical superheat, the heat flux reached the maximum and then reduced itself together with the superheat. This bubble-swelling without mobility is typical for the nucleation and growth stage of vapor bubbles in the conventional boiling heat transfer process.

On the other hand, as shown in Figure 8b, the center part of the micro-cavity-textured aluminum sheet was free of bubbles. Although larger bubbles swelled on the edges of the aluminum sheet, most of the tiny bubbles, with an average size of 0.01 mm or less, flowed together with the liquid channel flow, as shown in Figure 3, without swelling on the textured aluminum sheet surface. This observation proves that the micro-cavity-textured aluminum sheet becomes wet, even when the superheat is increased. 

**Figure 9:**
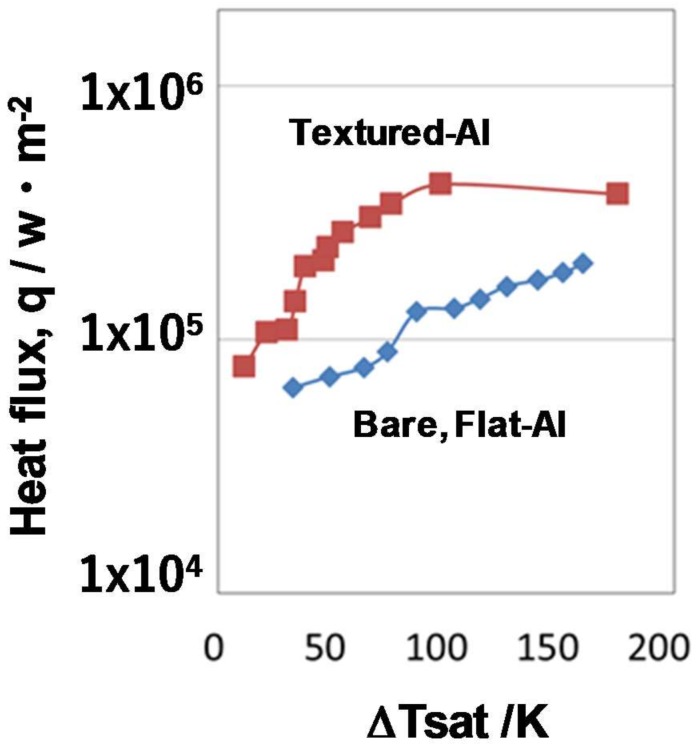
Heat transfer diagram between the measured heat flux and the superheat.

The heat flux, q, was also monitored by using measured temperatures T_1_ and T_2_ (<T_1_), at two different positions in the copper block with a distance of h, as depicted in Figure 3; i.e., q = (T_1_ − T_2_)/h. Figure 9 shows the boiling curve between the measured q and the superheat ΔT_sat_. The maximum heat flux for the bare and textured aluminum sheets did not reach a critical heat flux of 1 × 10^6^ W/m^2^, which is the theoretical estimate given in [18]. When ΔT_sat_ = 50 K, the heat flux became 3 × 10^5^ W/m^2^ on the micro-cavity-textured aluminum sheet, and became q = 7 × 10^4^ W/m^2^ on the bare, flat aluminum surface. The heat flux on the micro-cavity-textured aluminum was five times higher than that on the bare aluminum sheet. This implies that micro-textures on the heat-transferring solid surface have a significant influence on the heat transfer mechanism. Hence, micro-texturing on the metallic sheets can be performed to attain higher heat fluxes, even if the superheat is the same as that of sheets without such texturing, and to investigate the possibility of improving heat flux above the theoretical criticality in the conventional heat transfer process.

## 4. Conclusions

Thick DLC films coated on a SKD11 substrate represent the first stage in building various functional DLC structures. In this study, DLC-punch arrays were fabricated by plasma oxidation printing for a rectangular pillar with a head size of 3.8 μm × 4.0 μm and an average height of 8 μm. Under a plasma oxidation rate of 8 μm/h, plasma printing was terminated to selectively remove the unprinted DLC and to form this DLC-punch array on the SK11 substrate. Through a micro-embossing process via the CNC stamper, the rectangular micro-pillar texture on the DLC-punch array was transcribed onto the pure aluminum sheet as a rectangular micro-cavity texture with a bottom size of 3.2 μm × 3.2 μm, a wall thickness of 1.8 μm, and a depth of 7.5 μm. This micro-cavity-textured aluminum improved the boiling heat transfer. The tiny vapor bubbles nucleated on this textured surface and easily flowed away from the surface together with the liquid water in the channel. The nucleation and growth mechanism of the vapor bubbles on the heated solid aluminum were controlled by this surface micro-texturing. The heat flux on the micro-cavity-textured aluminum was five times higher than that on the bare, flat aluminum, even at the same superheat of 50 K.
